# Brain Atrophy Rates for Stable Multiple Sclerosis Patients on Long-Term Fingolimod versus Glatiramer Acetate

**DOI:** 10.3389/fneur.2020.01045

**Published:** 2020-09-23

**Authors:** Justin M. Honce, Kavita V. Nair, Brian D. Hoyt, Rebecca A. Seale, Stefan Sillau, Eric Engebretson, Brittany Schurr, John R. Corboy, Timothy L. Vollmer, Enrique Alvarez

**Affiliations:** ^1^Department of Radiology, University of Colorado Hospital, Aurora, CO, United States; ^2^Department of Clinical Pharmacy, University of Colorado, Aurora, CO, United States; ^3^Department of Neurology, University of Colorado and Rocky Mountain Multiple Sclerosis Center at the University of Colorado, Aurora, CO, United States; ^4^Department of Neurosurgery, University of Colorado, Aurora, CO, United States

**Keywords:** multiple sclerosis, brain atrophy, comparative effectiveness, fingolimod, glatiramer acetate, percent brain volume change

## Abstract

**Background:** Clinically stable multiple sclerosis (MS) patients on long-term therapy often have negligible acute inflammation on MRI. Brain atrophy may provide insight into subclinical disease progression in such populations.

**Objective:** This study aims to compare brain atrophy for age- and gender-matched MS patients treated for >2 years with fingolimod (FTY) or glatiramer acetate (GA), examining brain volume, cognition, and patient-reported outcomes (PROs).

**Methods:** Stable relapsing-MS patients, age 18–60, on FTY or GA for >2 years were followed up for 2 years. MRI brain and lesion volumes, cognitive measures, and PROs were collected at baseline and annually.

**Results:** Forty-four FTY and forty-three GA patients completed baseline and year 2 visits. No differences in age, gender, or education were observed. Median EDSS was 2.0_GA_ and 2.5_FTY_ (*p* = 0.22). Treatment duration was longer for GA, 6.50_GA_ vs. 3.73_FTY_ years (*p* < 0.001). Baseline geometric mean T2LV were different, GA = 1,009.29 cm^3^ vs. FTY = 2,404.67 cm^3^ (*p* = 0.0071). Baseline brain volumes were similar, GA = 1,508 cm^3^ vs. FTY = 1,489 cm^3^ (*p* = 0.2381). Annualized atrophy rates, adjusted for baseline and at mean baseline value, were GA = −0.2775% vs. FTY = −0.2967% (*p* = 0.7979). No differences in cognitive measures or PROs were observed.

**Conclusions:** Stable MS patients on long-term treatment with FTY and GA have similar brain volume loss rates. Differences in baseline disease severity may suggest patients with more aggressive disease treated with FTY may achieve similar brain volume loss rates as patients with milder baseline disease on GA.

## Introduction

Multiple sclerosis (MS) is characterized by a progressive and irreversible accumulation of injury to the central nervous system (CNS) (1). The primary goal of disease-modifying therapies (DMTs) remains the prevention of CNS injury (2). Classically, MRI measures used to quantify this CNS injury have focused on inflammatory white matter injury (3), specifically the accumulation of T1 hypointense, T2 hyperintense, and gadolinium-enhancing lesions in the white matter (4–6). While these have been the main focus of investigation, development of numerous semiautomated and automated techniques to quantify brain atrophy (7) have shown that brains of MS patients atrophy at a rate much faster than non-MS controls, and this atrophy appears to correlate more strongly with, and are better predictors of, clinical and cognitive disability than standard lesion-based metrics (8, 9).

Fingolimod (FTY) and glatiramer acetate can be considered “second”- and “first”-generation disease-modifying agents, respectively. FTY was the first oral DMT approved in 2010 and is a sphingosine-1-phosphate receptor modulator with demonstrated effects reducing the progression of disability, and the rate of brain atrophy, as well as the development of T2 and enhancing lesions (10, 11). GA is older, first approved for treatment in the USA in 1997 and has been shown to have an effect on relapses (12, 13), but the efficacy of GA is modest compared with several other presently used DMTs (14). These two DMTs have only recently been directly compared in the phase IIIB ASSESS trial which demonstrated that FTY 0.5 mg was superior to glatiramer acetate with a relative reduction in annualized relapse rate of 40.7%, *p* = 0.0138, a 54.5% relative reduction in mean number of new or enlarging lesions, and 55.6% relative reduction in gadolinium-enhancing T1 lesions compared with GA (15).

In various clinical trials comparing DMTs, it has been noted that there is a dynamic change in brain atrophy rates during the first 2 years of therapy, with greater atrophy rates seen in the first year after initiating therapy (16–18). This early volume loss is referred to as “pseudoatrophy” and is thought to represent a decrease in brain swelling as acute brain inflammation subsides (18). As such, for the majority of DMTs, the impact on brain atrophy is delayed until the second year, where the effects of pseudoatrophy are minimized.

Brain atrophy reduction in the first few years after initiation of DMT therapies is typically in the 40–50% range for highly effective therapies but has not normalized to rates seen in healthy populations (19). These studies are largely placebo-controlled studies and focus on the early years of treatment. It is currently unknown whether rates of atrophy in MS patients who have been stable on modern therapy long term (>2 years) differs between DMTs, and there are no published comparisons of atrophy rates in stable patients on FTY vs. GA. If there are differences in atrophy rates in this setting, it may indicate the persistence of subclinical disease activity and suggest that additional treatment strategies may be necessary to fully curb such disease-related atrophy.

This study examines whether there are differences in brain atrophy rates between RRMS patients who have been stable on long-term (>2 years) treatment with either FTY or GA, and whether there are differences in physical or cognitive impairment or patient-reported quality of life.

## Materials and Methods

This was a prospective observational between-group comparison study of MS patients on long-term FTY treatment (>2 years at enrollment), compared with age- and gender-matched patients on similar long-term GA therapy. Patients were followed up for 2 years, with imaging, clinical, and PRO data collected at baseline, at the end of the 1st year and end of the 2nd year of the study (see study flow diagram). Patients were first identified through chart review to determine if they met study inclusion criteria, following which they were contacted via phone or during regularly scheduled clinic visits for study participation. The study protocol was approved by the Colorado Multiple Institutional Review Board, and all study participants provided written informed consent before undergoing study procedures.

### Study Criteria

The study population was recruited from the Rocky Mountain MS Center at the University of Colorado in Aurora, Colorado. Inclusion criteria included patients between 18 and 60 years of age with relapsing MS as defined by the 2010 revised McDonald criteria (20). Subjects were required to be continuously taking FTY or GA for a minimum of 2 years prior to enrollment and to be able to provide written informed consent and comply with protocol requirements for the duration of the study.

Subjects were excluded from the study if they were suffering from comorbidities that, in the opinion of the investigators, could confound MRI outcomes (diabetes, stroke, etc.). Other exclusion criteria included the following: relapse or systemic steroid use within 2 years of the baseline visit, prior treatment with chemotherapy, cranial radiation or intracranial surgery, or if they were non-English speaking (as the PRO instruments were only validated in English at the time of the study). All female subjects were not pregnant or lactating and were required to practice an acceptable method of birth control during the study period.

### Image Acquisition and Processing

The first 11 patients (2 GA, 9 FTY) for the study were originally scanned on a 3.0-T Signa MRI scanner, after which this scanner was unexpectedly replaced by our institution with a 3.0-T Siemens Skyra MRI scanner. In order to ensure optimal consistency of imaging for this study, these patients were contacted to provide consent for an additional study visit to allow baseline, year 1, and year 2 imaging on an identical scanner for all participants. As such, for all patients included in this analysis, whole-brain MRI without gadolinium was performed on a 3.0-T Siemens Skyra MRI scanner as follows: (1) two-dimensional, dual-echo proton density and T2-weighted fast spin-echo images with the following parameters: 3 mm slice thickness, TE = min, TR = 2,600, ETL (2) two-dimensional fluid-attenuated inversion recovery fast spin-echo images with 3 mm slice thickness, (3) three-dimensional axial T1-weighted FSPGR images with 1 mm slice thickness and isotropic voxel diameter. All images were obtained with a 256 × 256 matrix size, with no interslice gaps.

An experienced neuroradiologist (JMH) and an image analyst, blinded to clinical details, identified hyperintense lesions on the proton density-weighted images, with reference to the T2-weighted and FLAIR images. This was used as a reference for semiautomated segmentation of lesions on the proton density-weighted images using the MS lesion finder tool in JIM 6.0 (Xinapse Systems). Errors in segmentation were manually corrected.

The resulting white matter lesion mask was used to infill lesions on the 3D T1-weighted images using the lesion_filling tool (21), part of the FMRIB Software Library (FSL; https://fsl.fmrib.ox.ac.uk/fsl/fslwiki). Automated brain extraction to remove the orbits and remaining non-brain tissues was performed using the brain extraction tool in FSL using optimized parameters (22). The brain-extracted, lesion-filled 3D T1 sequence from the baseline visit was used to quantify baseline normalized brain volume (NBV), and baseline, and year 2 three-dimensional T1 sequences were used to quantify global percent brain volume change (PBVC) over the 2 years of the study using SIENAX/SIENA (23, 24).

### Physical Disability Assessment

Physical disability was assessed at each study visit using the Expanded Disability Status Scale (EDSS) (25), as well as via the Patient-Determined Disease Steps (PDDS), a validated patient-reported outcome (PRO) version of the EDSS (26).

### Neuropsychological Testing

All patients underwent neuropsychological testing at each study visit (baseline, end of year 1, and after year 2 at the end of the study period). The individuals performing the testing were blinded to the MRI results. Neuropsychological tests from the MACFIMS battery were chosen to assess for deficits in information processing speed and memory. The following tests were conducted: (1) Wide Range Achievement Test-−4 Reading subtest, (2) Symbol Digit Modalities Test (SDMT, (3) Paced Auditory Serial Addition Test (PASAT), California Verbal Learning Test-II (CVLT-II), Brief Visuospatial Memory Test-Revised (BVMT-R), and the Controlled Oral Word Association Test (COWAT).

### Patient-Reported Outcome Measures

Patient-reported outcomes were assessed using the Neuro-Qol short forms, which allow for self-assessment of physical, mental, and social quality of life from these domains: *physical*: (1) upper extremity function, (2) lower extremity function, (3) fatigue, and (4) sleep disturbance; *mental*: (1) applied cognition—general concerns, (2) applied cognition—executive function, (3) communication, (4) anxiety, (5) depression, (6) emotional and behavioral dyscontrol, and (6) positive affect and well-being; and *social*: (1) satisfaction with social roles and activities and (2) ability to participate in social roles and activities.

### Statistical Analysis

#### Brain Volume/Lesion Characteristics

Baseline statistics for normalized brain volume, T2 lesion volume, and number of T2 lesions for the entire sample and by treatment group were determined including mean, standard deviations, medians, and interquartile range. Since raw T2 lesion volumes were strongly right skewed (and there are no zeros), a logarithmic transformation was used for analysis.

#### Percent Brain Volume Change

PBVC from baseline to year 2 was determined by comparing paired MRI scans. Regression analysis has been adjusted for baseline brain volume. Estimated mean changes by group, for the mean value of baseline brain volume, and the mean difference between groups were calculated; 95% confidence intervals were included, and *p*-values tested the null hypotheses of no change within group and no difference in change between groups. Univariate *T* statistics were used for inference. Another regression analysis compared PBVC between groups, controlled for baseline disease duration, treatment duration, and the T2 lesion volume on logarithmic scale. Different residual variances were allowed by treatment group, and the Satterthwaite method was used for degrees of freedom. Statistical analyses for T2 lesion volume were performed on the logarithmic scale because the distribution of T2 lesion volumes was strongly right skewed, and a logarithmic transform renders the distribution more Gaussian. Back transforming means on the logarithmic scale yields geometric means, and back transforming differences on the logarithmic scale yields ratios.

#### Neuropsychological Assessments and Patient-Reported Outcomes

Means and standard deviations for the neuropsychological tests and each of the 13 Neuro-QoL scales were summed at baseline for the entire sample and within treatment group. Between group mean differences, and their *p*-values for the null hypothesis of no mean differences were estimated. The *p*-values were calculated using the standard two-sample Satterthwaite *t*-test for neuropsychological tests and the one sample test for PROs. Change scores were derived for both neuropsychological and PROs comparing baseline to year 2 and were correlated with PBVC for year 2 using the Spearman's rank method.

## Results

### Recruitment and Study Flow

**Figure 1:**
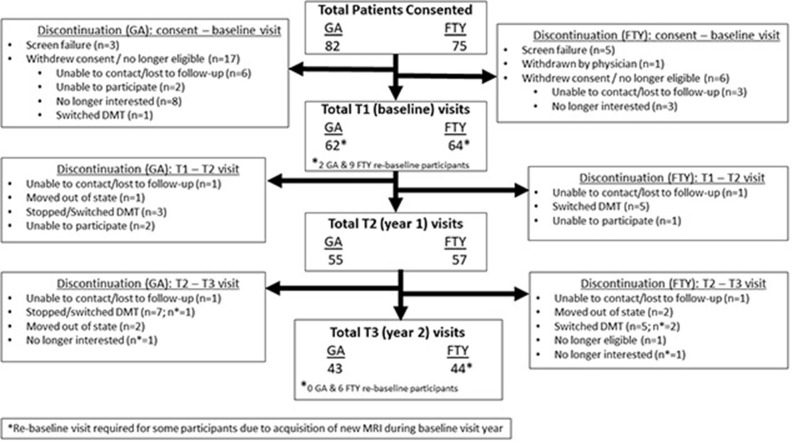
Recruitment and flow chart of study subjects.

Recruitment occurred over an 18-month period as shown in Figure 1. A total of 82 subjects for GA group and 75 for the FTY group were screened. Sixty-two GA subjects and 63 FTY subjects completed their baseline visits. Fifty-five GA subjects and 57 FTY subjects completed their year 1 visit, and 43 GA subjects and 44 FTY subjects completed their final study visit. Reasons for withdrawal after the baseline visit varied are detailed in Figure 1.

### Baseline Characteristics

As seen in Table 1, there were no statistically significant differences between the GA and FTY subjects for age, gender, ethnicity, or race. MS disease duration was similar, but subjects on GA had longer treatment durations (mean, 6.5 years for GA and 3.7 years for FTY; *p* = 0.0004). No differences were found in physical disability as measured by the EDSS or PDSS. Average EDSS scores across all groups was 2.00 (range, 1.50–3.00) at baseline (*p* = 0.2163). There were similar types of comorbidities in each group, with the only statistically significant differences seen for pulmonary conditions (10 in the FTY group and eight in the GA group; *p* = 0.0492). Living status was also similar between groups.

**Table 1:** Demographic and MS disease characteristics of the study population.

	**ALL (*n* = 87)**	**GA (*n* = 43)**	**Fingolimod (*n* = 44)**	**Mean difference estimate (FTY vs. GA)**	***p*-value**
Baseline characteristics					
Age	49.60 (±7.60)	49.85 (±7.69)	49.36 (±7.59)	−0.4897	0.7658
Gender (% female)	69 (79.31%)	34 (79.07%)	35 (79.55%)	0.0048	0.9563
Years of education	13.68 (±1.40)	13.77 (1.46)	13.59 (1.35)	−0.1765	0.5604
Ethnicity					1.0000
Hispanic	7 (8.05%)	3 (3.98%)	4 (9.09%)	0.0211	
Non-hispanic	79 (90.80%)	39 (90.70%)	40 (90.91%)	0.0021	
Unknown	1 (1.15%)	1 (2.33%)	0 (0.00%)	−0.0233	
Race					
% Caucasian	77 (88.51%)	39 (90.70%)	38 (86.36%)	−0.0433	0.7387
% African American	2 (2.30%)	1 (2.33%)	1 (2.27%)	−0.0005	1.0000
% Native American	1 (1.15%)	0 (0.00%)	1 (2.27%)	0.0227	1.0000
% Asian/Pacific Islander	1 (1.15%)	1 (2.33%)	0 (0.00%)	−0.0233	0.4943
% Hispanic	7 (8.05%)	3 (6.98%)	4 (9.09%)	0.0211	1.0000
% Other	1 (1.15%)	1 (2.33%)	0 (0.00%)	−0.0233	0.4943
MS disease characteristics					
MS disease duration	12.05 (±5.61)	10.98 (±6.58)	13.09 (±4.29)	2.1142	0.0808
Treatment duration	5.10 (±3.58)	6.50 (±4.55)	3.73 (±1.21)	−2.7633	0.0004
EDSS	2.00 (1.50–3.00)	2.00 (1.50–3.00)	2.50 (2.00–3.00)	0.5	0.2163
PDDS	0.00 (0.00–1.00)	0.00 (0.00–1.00)	0.00 (0.00–1.50)	0	0.5473
Smoking status:					0.0833
Current smoker	4 (4.60%)	0 (0.00%)	4 (9.09%)	0.0909	
Past smoker	29 (33.33%)	13 (30.23%)	16 (36.36%)	0.0613	
Never smoker	54 (62.07%)	30 (69.77%)	24 (54.55%)	−0.1522	
High cholesterol	14 (16.09%)	4 (9.30%)	10 (22.73%)	0.1342	0.0884
High blood pressure	15 (17.24%)	7 (16.28%)	8 (18.18%)	0.019	1.0000
Lung trouble	10 (11.49%)	8 (18.60%)	2 (4.55%)	−0.1406	0.0492
Diabetes	1 (1.15%)	1 (2.33%)	0 (0.00%)	−0.0233	0.4943
Migraines	18 (20.69%)	10 (23.26%)	8 (18.18%)	−0.0507	0.5591
Thyroid issues	13 (14.94%)	7 (16.28%)	6 (13.64%)	−0.0264	0.7296
Degenerative arthritis	3 (3.45%)	2 (4.65%)	1 (2.27%)	−0.0238	0.6162
Osteoporosis	2 (2.30%)	2 (4.65%)	0 (0.00%)	−0.0465	0.2414
Irritable bowel syndrome	4 (4.60%)	1 (2.33%)	3 (6.82%)	0.0449	0.6162
Depression	26 (29.89%)	17 (39.53%)	9 (20.45%)	−0.1908	0.0519
Anxiety	11 (12.64%)	6 (13.95%)	5 (11.36%)	−0.0259	0.7163
Living status (SUPPL)					
Alone	18 (20.69%)	11 (25.58%)	7 (15.91%)	−0.0967	0.2655
Spouse/partner	64 (73.56%)	29 (67.44%)	35 (79.55%)	0.121	0.2006
Children	33 (37.93%)	16 (37.21%)	17 (38.64%)	0.0143	0.8909
Parent	1 (1.15%)	0 (0.00%)	1 (2.27%)	0.0227	1.0000
Sibling	1 (1.15%)	0 (0.00%)	1 (2.27%)	0.0227	1.0000
Other relative	1 (1.15%)	0 (0.00%)	1 (2.27%)	0.0227	1.0000
Friend/companion	1 (1.15%)	1 (2.33%)	0 (0.00%)	−0.0233	0.4943
Domestic help (caregiver)	0 (0.00%)	0 (0.00%)	0 (0.00%)	0	

At baseline, T2 lesion volumes were different between groups, with the T2 lesion volumes measuring substantially larger in the FTY group (2,617 mm^3^) vs. in the GA group (860 mm^3^), *p* = 0.0102. Despite the substantially greater severity in lesion burden in the FTY group, normalized brain volumes were similar (1,489 cm^3^ for FTY vs. 1,508 cm^3^ for GA; *p* = 0.2381). At baseline, no differences were found between groups for any of the neuropsychological measures or Neuro-QoL PROs.

### Longitudinal Characteristics

Mean normalized brain volume at baseline, for both groups pooled, was 1,498.56 cm^3^. Whole brain atrophy, as measured as the estimated mean annualized PBVC over the 2-year study period, for the mean baseline brain volume, was −0.2775% in the GA group (95% CI, −0.3752, −0.1801%, *p* < 0.0001) and −0.2967% in the FTY group (95% CI, −0.4109, −0.1827%; *p* < 0.0001). These rates were not statistically different between groups, *p* = 0.7979. A regression analysis, controlled for baseline brain volume, disease duration, treatment duration, and the T2 lesion volume on logarithmic scale, estimated the difference in annualized PBVC between FTY and GA as −0.1023% (95% CI, −0.1797, 0.2001%; *p* = 0.9149) (Note: annualized with the Taylor series method). At the mean values of the covariates, the estimated annualized mean PBVC was −0.2821% (95% CI, −0.3973, −0.1669%; *p* < 0.0001) in the GA group and −0.2923% (95% CI: −0.4203, −0.1645%; *p* < 0.0001) in the FTY group.

No statistical differences exist in T2 lesion volume over the study period (Table 2). A regression analysis, which controlled for baseline disease duration, treatment duration, and the T2 lesion volume on logarithmic scale, estimated the ratio between FTY and GA of the annualized change ratios of T2 lesion volume as 0.9882 (95% CI, 0.9277, 1.0526; *p* = 0.7096). At the mean values of the covariates, the estimated mean annualized change ratio was 1.0526 (95% CI, 1.0089, 1.0981; *p* = 0.0188) in the GA group and 1.0402 (95% CI, 1.0007, 1.0812: *p* = −0.0463) in the FTY group.

**Table 2:** Percent brain volume change.

	**ALL (*n* = 87)**	**GA (*n* = 43)**	**Fingolimod (*n* = 44)**	***p*-value**
Baseline MRI measures	Mean (95% CI)			
Normalized brain volume (cm^3^)	1,498.56 (1,482.94, 1,514.18)	1,507.97 (1,486.28, 1,529.66)	1,489.36 (1,466.38, 1,512.34)	0.2381
Raw T2 lesion volume (mm^3^) (medians)	1,674.52 (1,142.78, 3,163.94)	859.87 (611.80, 1,888.83)	2,616.87 (1,558.07, 4,541.55)	0.0102
T2 lesion volume (mm^3^) (geometric means)	1,565.67 (1,132.58, 2,164.38)	1,009.29 (628.43, 1,620.96)	2,404.67 (1,577.82, 3,664.81)	0.0071
Longitudinal MRI measures				
Annualized PBVC (%) [baseline-normalized brain volume = 1,498.56 (cm^3^)]	−0.2873 (−0.3607, −0.2139)	−0.2775 (−0.3752, −0.1801)	−0.2967 (−0.4109, −0.1827)	0.7979
Annualized change in T2 lesion volume (ratio of geometric means) [baseline T2 lesion volume = 1,565.67 (mm^3^)]	1.0463 (1.0203, 1.0729)	1.0392 (0.9992, 1.0807)	1.0533 (1.0173, 1.0906)	0.6119

Scores from the PRO assessments did not significantly change over the study period. However, there were improvements on some neuropsychological variables over 2 years, and no significant decreases on neuropsychological variables were observed (Table 3). No differences were seen between GA and FTY in neuropsychological variables and PROs from baseline to year 2. There were no notable correlations between PBVC, neuropsychological variables, and the PROs.

**Table 3:** Baseline neuropsychological and PRO scores.

	**GA change baseline to year 2**				**FTY change baseline to year 2**				**Group difference change**		
**Variable**	**GA mean**	**GA standard error**	***T* statistics**	***p*-value**	**FTY mean**	**FTY standard error**	***T* statistics**	***p*-value**	**Mean difference estimate**	***T* statistic**	***p*-value**
PASAT 2 sec.	3.3488372	0.8298712	4.04	**0.0002**	3.8372093	1.0976317	3.5	**0.0011**	−0.4884	−0.35	0.7236
PASAT total	7.372093	1.3231993	5.57	**<0.0001**	6	1.7240402	3.48	**0.0012**	1.3721	0.63	0.5296
SDMT oral	2.255814	1.0701285	2.11	**0.041**	−0.255814	0.8320098	−0.31	0.76	2.5116	1.85	0.0676
SDMT written	0.0232558	0.0232558	1	0.323	0.1162791	0.0494634	2.35	**0.0235**	−0.093	−1.7	0.094
CVLT-II trials 1–5	5.4186047	1.0743318	5.04	**<0.0001**	5.8139535	1.2425886	4.68	**<0.0001**	−0.3953	−0.24	0.8104
CVLT-II LD free recall	1.0232558	0.3385337	3.02	**0.0043**	0.6976744	0.3353233	2.08	**0.0436**	0.3256	0.68	0.4963
BVMT-R trials 1–3	1.9767442	0.6491227	3.05	**0.004**	1.6744186	0.6294023	2.66	**0.011**	0.3023	0.33	0.7389
BVMT-R delayed recall	0.3023256	0.2567686	1.18	0.2457	0.5581395	0.2189837	2.55	**0.0146**	−0.2558	−0.76	0.4506
D-KEFS letter fluency	0.3953488	1.321154	0.3	0.7662	3.9302326	1.1524904	3.41	**0.0014**	−3.5349	−2.02	**0.047**

*Statistically significant p-values were indicated by making the text bold*.

Average EDSS scores did not change over the study period: 0.09 for GA and −0.16 for FTY, *p* = 0.2721 (Table 4).

**Table 4:** Change in disability scores.

	**ALL (*n* = 87)**	**GA (*n* = 43)**	**Fingolimod (*n* = 44)**	***p*-value**
2-year change in mean EDSS (imputed baseline value for re-baseline patients)	−0.02 (−0.24, 0.19) (*N* = 81)	0.09 (−0.14, 0.32) (*N* = 43)	−0.16 (−0.56, 0.24) (*N* = 38)	0.2721
2-year change in mean PDDS	0.03 (−0.14, 0.19)	0.10 (−0.10, 0.30) (*N* = 40)	−0.05 (−0.33, 0.22) (*N* = 37)	0.3600

## Discussion

Our results suggest that stable RRMS patients who have been maintained on long-term GA or FTY therapies demonstrate relatively modest rates of brain volume loss over 2 years, and that the rate of this atrophy is similar between groups.

As expected, the measured atrophy rates for FTY are lower than previously reported atrophy rates from earlier in treatment; for instance, *post-hoc* analysis of the phase 3 FREEDOMS/FREEDOMS II studies showed that during the first 2 years, unadjusted and adjusted atrophy rates for FTY ranged from −0.79 to 0.91% (10). This difference is likely to be at least partly related to the pseudoatrophy effect seen after initiation of FTY, as acute neuroinflammation subsides during the early years of therapy. Furthermore, in the same study, a subgroup analysis of those patients with no baseline enhancing lesions and no relapses or new lesions during the study (i.e., those with the mildest disease and least likelihood to have significant acute neuroinflammation), the atrophy rates were more in line with the rates reported in our study, even somewhat lower, likely related due to selection of those with the mildest disease (10). The rates of atrophy reported from the MS-MRIUS study of real-world follow-up of patients treated with FTY also showed similar results. Specifically, Zivadinov et al. (27) reported that the rate of PBVC in patients with no evidence of active disease (most similar to the patients in our group) was −0.25%, which is very similar to our reported annual atrophy rate in the FTY arm.

In the long-term follow-up extension study of patients treated with GA from the European/Canadian double-blinded, placebo-controlled MRI-monitored trial, the 4.3-year atrophy rate measured from 18 months after initiation of therapy was −3.32%, which is ~0.77%/year (28). Of note, the T2 lesion burden in those patients was substantially greater than in our GA subjects, suggesting that this difference may relate to differences in overall severity of disease burden. In another study comparing patients treated with GA or interferon over 5 years, with lesion burdens still greater than for GA subjects in our study, but much less than in the European/Canadian trial, atrophy rates were smaller, ~0.45%/year (29).

In their attempt to establish pathological cut-offs for brain atrophy rates in multiple sclerosis patients, De Stefano and colleagues have proposed that an annual PBVC/year of 0.4% may best differentiate between normal and abnormal atrophy rates (30). With this cut-off in mind, our results suggest that atrophy rates in both groups may have normalized (0.2881 PBVC/year for GA and 0.2901 PBVC/year for FTY), perhaps indicating resolution of subclinical disease progression.

In our study population, a key point to note is that study subjects treated with FTY demonstrated substantially larger T2 lesion volumes than those in the group treated with GA. This difference may be attributed to the prescription practices of the MS neuroimmunologists at the Rocky Mountain MS Center, who favor initiation of highly effective therapies such as FTY as first-line treatment choices in appropriate patient populations. In our practice, patients are maintained on GA only if (1) that is the patient's preference, (2) there is no evidence of clinical or lesional MRI disease progression, or (3) due to pregnancy. As such, it is rare for patients with moderate or severe MS to be maintained on GA in our practice. Despite these differences in severity of lesion burden, baseline-normalized brain volumes and atrophy rates are similar between groups in this study, which suggests that FTY may slow the rate of brain volume loss more effectively than GA.

Despite baseline differences in T2 lesion volumes, there were no differences in cognitive functioning between patients treated with FTY and patients treated with GA. Additionally, cognitive functioning did not decline over 2 years in either group and a few improvements were observed, likely attributable to practice effects. In accordance, no group differences in change on any of the neuropsychological variables were observed. The lack of difference between groups in baseline cognitive performance and change over 2 years parallels the finding regarding brain atrophy and suggests that FTY may slow the rate of brain atrophy more effectively and preserve cognitive function in patients with a higher lesion volume in comparison with patients with milder disease maintained on GA. Given the lack of differences between groups for the neuropsychological tests, it is perhaps unsurprising that there were no differences in either group for self-report scales of physical, mental, and social quality of life at baseline or over 2 years.

There are several limitations in this study. While both the FTY and GA groups in our study were matched for age and gender, given the prescription practices at our institution favoring GA only for stable patients with mild disease or when pregnant, the FTY group had substantially greater baseline disease burden as evidenced by the significantly greater T2 lesion burden in this group. Unfortunately, matching the study groups for lesion burden was not feasible for this study, but even when statistically controlling for these differences, atrophy rates remained non-significantly different between groups. Given the small sample size of our study and resultant limited power, it is possible that a larger study may show evidence of differences in atrophy rates between groups. The atrophy results for the recently completed ASSESS trial will be of particular interest in this regard (31).

The results of this study show that RRMS patients who can remain stable on disease-modifying therapies such as GA and FTY for many years have relatively low rates of brain atrophy, and that these rates of atrophy are below cut-offs previously reported as discriminating between normal and abnormal subjects. Additionally, despite significantly greater T2 lesion burdens in the patients treated with FTY, brain volumes, atrophy rates, and levels of clinical and cognitive impairments were similar across both groups.

## Data Availability Statement

The datasets generated for this article are not readily available because the data is a unique combination of four sets of combined analytical data from our center: MRI, patient reported outcomes, abridged MACFIMS, and clinical MS disease history with patient demographics. Providing this dataset would risk the possibility of breaching patient confidentiality as well as the proprietary nature of our combined dataset.

## Ethics Statement

The study involving human participants was reviewed and approved by Colorado Multiple Institutional Review Board. The patients/participants provided their written informed consent to participate in this study.

## Author Contributions

JH, KN, EA, JC, BH, SS, and TV contributed to project conception, data collection, data analysis, and manuscript editing. BH, EE, and BS contributed to data collection, analysis, and manuscript editing. RS contributed to data analysis and manuscript editing. All authors contributed to the article and approved the submitted version.

## Conflict of Interest

JH has received compensation for consultancy from Genentech and Medtronic and research grants from Novartis and Biogen. KN has received compensation for consultancy from Celgene, Novartis, Genentech, and EMD Serono and grants from Genentech, Novartis, and BMS. JC has received compensation for activities such as advisory boards, lectures, and consultancy with the following companies and organizations: Mylan, Novartis, Prime CME, and Rocky Mountain MS. He has received research support from the following: MedDay, Novartis, National MS Society, and Patient Centered Outcomes Research Initiative (PCORI). TV has received compensation for lectures and consultancy with the following: Biogen IDEC, Genentech/Roche, Siranax, Celgene, EMD Serono, and Novartis. He has received research support from the following: Rocky Mountain MS Center; Biogen; Actelion; Roche/Genentech; F. Hoffman-La Roche, Ltd, and TG Therapeutics, Inc. EA has received compensation for activities such as advisory boards, lectures, and consultancy with the following companies and organizations: Actelion/Janssen, Bayer, Biogen, Celgene, EMD Serono, Genentech, Genzyme, Novartis, and TG Therapeutics. He has received research support from the following: Biogen, Genentech, Novartis, TG Therapeutics, Patient Centered Outcomes Research Initiative (PCORI), and Rocky Mountain MS Center. The remaining authors declare that the research was conducted in the absence of any commercial or financial relationships that could be construed as a potential conflict of interest.
